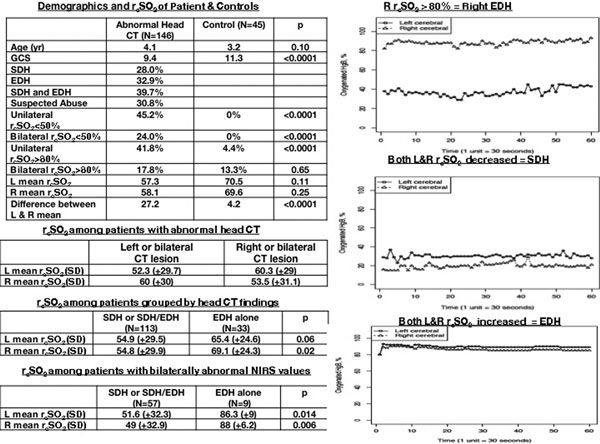# Cerebral rSO_2 _monitoring in pediatric altered mental status patients

**DOI:** 10.1186/cc12266

**Published:** 2013-03-19

**Authors:** T Abramo, I Kane

**Affiliations:** 1 Vanderbilt School of Medicine, Nashville, TN, USA

## Introduction

Pediatric patients with altered mental status (AMS) present with poor histories resulting in delayed testing and potential poor outcomes. Non-invasive detection for altered cerebral physiology related to TBI would improve resuscitation and outcome. Cerebral rSO_2 _(r_c_SO_2_) studies demonstrate its utility in certain neurological emergencies.

## Methods

A retrospective analysis of r_c_SO_2 _utility in AMS. rcSO_2 _data were collected every 30 seconds for AMS patients who had a head CT. Patients with a negative head CT were compared with those with an abnormal head CT. ROC analysis was performed to find the AUC for each summary statistic and performance characteristics. Subgroup analysis was done to determine whether r_c_SO_2 _predicted injury and location.

## Results

r_c_SO_2 _readings across 5, 15, 20, and 30 minutes were stable (Figure 1). r_c_SO_2 _readings with one or both sides <50% or a wide difference between L and R cerebrum was predictive of an abnormal CT scan. A mean difference of 4.2 was 82% sensitive for detecting a CT lesion with 62% specificity, 88% PPV, and 52% NPV; a mean difference of 12.2 was 100% specific for an abnormal head CT. Lower mean r_c_SO_2 _readings localized to the CT pathology side, and higher r_c_SO_2 _readings trend toward the EDH group.

## Conclusion

Cerebral rcSO_2 _monitoring can non-invasively detect altered cerebral physiology and pathology related to TBI as the cause for pediatric altered mental status. The utility of r_c_SO_2 _monitoring has shown its potential for localizing and characterizing intracranial lesions among these altered children. Further studies utilizing r_c_SO_2 _monitoring as an adjunct tool in pediatric altered mental status evaluation and management are ongoing.

**Figure 1:**